# Benefits of lumbar spine fusion surgery reach 10 years with various surgical indications

**DOI:** 10.1016/j.xnsj.2023.100276

**Published:** 2023-09-09

**Authors:** Leevi A. Toivonen, Arja Häkkinen, Liisa Pekkanen, Kati Kyrölä, Hannu Kautiainen, Marko H. Neva

**Affiliations:** a Department of Orthopedics and Traumatology, Tampere University Hospital, Elämänaukio 2, PB 272, Tampere, 33101, Finland; b Faculty of Sport and Health Sciences, University of Jyväskylä, Seminaarinkatu 15, Jyväskylä, 40014, Finland; c Department of Surgery, Central Finland Healthcare District, Hoitajantie 3, Jyväskylä, 40620, Finland; d Primary Health Care Unit, Kuopio University Hospital, Yliopistonranta 8, Kuopio, 70210, Finland; e Folkhälsan Research Center, Topeliuksenkatu 20, Helsinki, 00250, Finland

**Keywords:** Lumbar spine fusion, Long-term, Outcome, Patient reported outcomes, PROM, Indication

## Abstract

**Background Context:**

Lumbar spine fusion (LSF) surgery is a viable form of treatment for several spinal disorders. Treatment effects are preferably to be endorsed in real-life settings.

**Methods:**

This prospective study evaluated the 10-year outcomes of LSF. A population-based series of elective LSFs performed at 2 spine centers between January 2008 and June 2012 were enrolled. Surgeries for tumor, acute fracture, or infection, neuromuscular scoliosis, or postoperative conditions were excluded. The following patient-reported outcome measures (PROMs) were collected at baseline, and 1, 2, 5, and 10 years postsurgery: VAS for back and leg pain, ODI, SF-36. Longitudinal measures of PROMs were analyzed using mixed-effects models.

**Results:**

A total of 683 patients met the inclusion criteria, and 630 (92%) of them completed baseline and at least 1 follow-up PROMs, and they constituted the study population. Mean age was 61 (SD 12) years, 69% women. According to surgical indication, patients were stratified into degenerative spondylolisthesis (DS, n=332, 53%), spinal stenosis (SS, n=102, 16%), isthmic spondylolisthesis (IS, n=97, 15%), degenerative disc disease (DDD, n=52, 8%), and deformity (DF, n=47, 7%).

All diagnostic cohorts demonstrated significant improvement at 1 year, followed by a partial loss of benefits by 10 years. ODI baselines and changes at 1 and 10 years were: (DS) 45, −21, and −14; (SS) 51, −24, and −13; (IS) 41, −24, and −20; (DDD) 50, −20, and −20; and (DF) 50, −21, and −16, respectively. Comparable patterns were seen in pain scores. Significant HRQoL achievements were recorded in all cohorts, greatest in physical domains, but also substantial in mental aspects of HRQoL.

**Conclusions:**

Benefits of LSF were partially lost but still meaningful at 10 years of surgery. Long-term benefits seemed milder with degenerative conditions, reflecting the progress of the ongoing spinal degeneration. Benefits were most overt in pain and physical function measures.

## Background

Lumbar spine fusion (LSF) surgery is an established method in the treatment of several spinal disorders. Its short-term efficacy has been demonstrated in various populations and distinct pathologies [1], [2], [3], [4], [5]. Great share of these surgeries, however, is carried out for degenerative causes, usually presenting with stenosis [6]. Therein, decompression bears an integral part of fusion surgery.

Effects of LSF predominantly consist of pain relief and functional gain resulting in improving health-related quality of life (HRQoL). It has been stated that LSF may grant short-term benefits at the expense of late-term consequences [7]. Occurrence of adjacent segment disease is a major jeopardizer of long-term treatment effect. It may necessitate repeat surgeries, which in turn may at least partially restore the earlier gains [8]. Preservation of the treatment effect is prerequisite for cost-effectiveness and thereby the overall rationale of the method [9].

Most long-term reports of LSF focus on select pathologies or compare treatment modalities [10], [11], [12], [13]. In addition, low response rates encumber many long-term follow-ups [14]. Reports of nonselected, real-life surgical populations are needed to widen perspectives on long-term efficacy of LSF.

In this study, we sought to scrutinize the 10-year outcomes of LSF on pain, disability, and HRQoL across different indications for elective fusion surgery. We hypothesized that the previously reported early benefits of LSF were substantially maintained at a longer follow-up.

## Methods

### Subjects and surgeries

This study is based on a series of elective LSF surgeries performed in 2 Finnish spine centers (Tampere University Hospital and Jyväskylä Central Hospital) between January 2008 and June 2012. These public hospitals represent an academic institution and a big central hospital, covering both urban and rural communities of 775,000 inhabitants. Due to Finland's national healthcare insurance system, at the data collecting period, all LSF surgeries for this population were performed in these centers. As a result, the study population also features a population-based sample of elective LSF surgeries.

At the outpatient clinic following decision on elective LSF, patients were offered enrollment in a prospective follow-up study. The attending surgeon procured a written, informed consent. Patients with tumor, acute fracture, or infection, neuromuscular scoliosis, and postoperative condition as an indication for surgery were not included.

The surgeon recorded surgical indications and, accordingly, classified patients into degenerative spondylolisthesis (DS), spinal stenosis (SS), isthmic spondylolisthesis (IS), degenerative disc disease (DDD), and deformity (DF). Spinal stenosis and DS cohorts feature spinal stenosis, DS with and SS without DS. Degenerative disc disease patients typically feature severe disc degeneration, frequently with disc bulging and radiculopathy yet without distinct stenosis in magnetic resonance imaging. The deformity cohort features spinal stenosis with degenerative scoliosis, kyphosis, or lateral spondylolisthesis.

Study setting had no interference to surgeries which were carried out according to standard clinical practice. Indications for surgery were similar across both centers. All surgeries were performed by 7 surgeons with experience of 5 to over 10 years in spine surgery. Also, all surgeons had operated together to ensure uniformity of procedures within and between centers. All surgeries were performed through open, posterior midline incision using pedicle screws. Open decompression was always performed in the presence of stenosis. Interbody spacers were used at the surgeons’ discretion. Ethical boards of both study centers had approved the study.

### Outcome measures

Treatment effect was evaluated using established patient-reported outcome measures (PROMs). Pain intensity was quantified with visual analogue scale (VAS) (0–100 mm) for back and leg (radicular) pain. Back-related disability was determined using the Finnish validated version 2.0 of the Oswestry Disability Index (ODI) [15,16]. Oswestry Disability Index score ranges between 0 and 100 with higher scores indicating higher levels of disability. Health-related quality of life (HRQoL) was measured with Short-Form 36 (SF-36) [17]. It has 8 domains that can be aggregated to physical (PCS) and mental (MCS) component summary scores. In the process, PCS is positively impacted by physical functioning, role physical, bodily pain, and general health domains and negatively impacted by mental health, vitality, social functioning, and role-emotional domains. Domains impact contrariwise with MCS. Each domain and summary scores range between 0 and 100, higher scores indicating better health. Patients’ baseline status was collected prior surgery, and follow-up data were collected at 1, 2, 5, and 10 years. A reminder letter was sent in a case of missing answers. Questionnaires were administered by study nurses, while postoperative follow-up visits at spine centers occurred at 3 months and 1 year postsurgery.

### Statistics

Data are presented as means with standard deviations (SD), medians with interquartile ranges (IQR), or frequencies with percentages. Differences in baseline demographical and clinical data across surgical indications (groups) were compared with one-way ANOVA, Kruskal–Wallis test, chi-square based test, or Fisher-Freeman exact test, as appropriate. Longitudinal measures of PROMs were analyzed using mixed-effects models with an unstructured covariance structure (ie, the Kenward–Roger method for calculating degrees of freedom). We consider fixed effects to include indication for surgery, time, and indication for surgery * time interactions. As the use of mixed models allows for analysis of unbalanced (eg, missing measurements) datasets without imputation, we analyzed all available data, using the full analysis set. Normal distributions were evaluated graphically, and with the Shapiro–Wilk W test. All analyses were performed with Stata 17.0 (StataCorp LP).

## Results

**Fig. 1:**
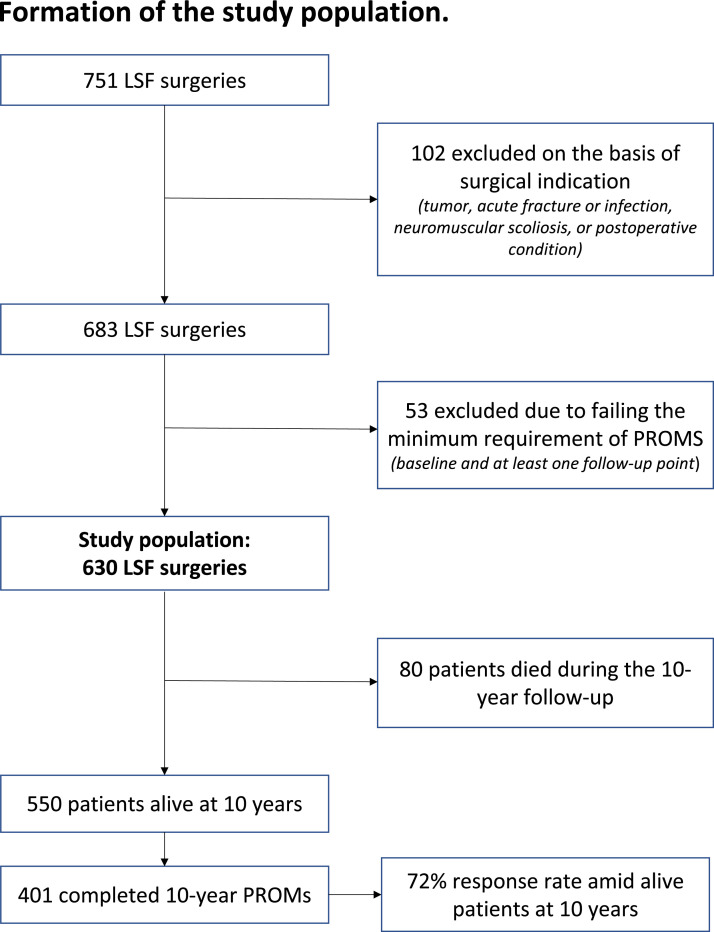
Formation of the study population.

During the data collecting period, 795 LSFs were performed in the 2 study centers. Before surgery, only 10 patients declined to participate. A total of 683 patients met the inclusion criteria (Fig. 1). Of those, 630 patients (92%) completed PROMs at baseline and at least 1 follow-up point, and they constituted the study population. At baseline, mean age of subjects was 61 (SD 12) years, 69% women. Demographical and clinical data are outlined in Table 1. A total of 80 (13%) participants died during the 10-year follow-up. A total of 440 subjects completed 10-year PROMs ensuing a 10-year response rate of 72% among then-alive study participants. Ten-year responses covered at least 63% of all performed surgeries meeting inclusion criteria (eligibility of the 10 refusers was not known).

**Table 1:** Baseline patient demographical and clinical data according to indication for surgery.

	DS N=332	SS N=102	IS N=97	DDD N=52	DF N=47	p-value
Women, n (%)	266 (80)	65 (64)	44 (45)	24 (46)	32 (68)	<.001
Age, mean (SD)	65 (10)	65 (9)	48 (10)	50 (13)	66 (7)	<.001
BMI, mean (SD)	28.1 (4.4)	29.0 (4.4)	27.3 (4.0)	26.8 (3.9)	27.4 (4.5)	.011
Education years, mean (SD)	11.4 (3.6)	11.5 (4.0)	12.9 (3.5)	13.9 (3.8)	10.2 (2.9)	.049
Cohabiting, n (%)	204 (62)	66 (67)	76 (78)	38 (75)	30 (64)	.024
Smoking, n (%)	39 (12)	15 (15)	28 (29)	14 (27)	4 (9)	<.001
Retired, n (%)	226 (68)	70 (71)	14 (14)	11 (22)	35 (74)	<.001
Comorbidities, n (%)						
Cardiovascular	200 (60)	55 (54)	30 (31)	18 (35)	29 (62)	<.001
Diabetes	39 (12)	17 (17)	7 (7)	4 (8)	8 (17)	.18
Rhematoid	39 (12)	12 (12)	1 (1)	1 (2)	10 (21)	<.001
Neurological	9 (3)	4 (4)	4 (4)	0 (0)	1 (2)	.65
Psychiatric	12 (4)	3 (3)	4 (4)	3 (6)	0 (0)	.62
Pulmonary	11 (3)	3 (3)	3 (3)	1 (2)	0 (0)	.90
Cancer	7 (2)	0 (0)	1 (1)	1 (2)	1 (2)	.56
Duration of symptoms, years, median (IQR)	10 (3, 20)	10 (6, 20)	10 (5, 20)	7 (3, 15)	10 (3, 22)	.074
Predominant symptom, n (%)						<.001
Radicular pain	246 (75)	62 (63)	55 (59)	26 (52)	20 (43)	
Back pain	45 (14)	21 (21)	36 (38)	21 (42)	22 (47)	
Lower limb weakness	36 (11)	16 (16)	3 (3)	3 (6)	5 (11)	
Surgery						
Length of fusion, levels (%)						<.001
1–2	257 (77)	41 (40)	91 (94)	35 (67)	15 (33)	
>2	75 (23)	61 (60)	6 (6)	17 (33)	31 (67)	
Interbody fusion, n (%)	39 (12)	4 (4)	58 (60)	14 (27)	10 (21)	<.001

DS, degenerative spondylolisthesis; SS, spinal stenosis; IS, isthmic spondylolisthesis; DDD, degenerative disc disease; DF, deformity

SD, standard deviation; IQR, interquartile range; BMI, body mass index.

According to surgical indication, patients distributed as follows: DS: (n=332, 53%); SS: (n=102, 16%); IS (n=97, 15%); DDD (n=52, 8%); and DF (n=47, 7%). Hence, 434 (69%) patients encompassed spinal stenosis (DS + SS). DDD and IS patients were younger and better educated compared to other cohorts. Patients with DS, IS, and DDD most often received short fusions (1-2 levels), whereas SS (without spondylolisthesis), and deformities ensued longer fusions pertaining to wider-ranging pathology. Rate of interbody spacer was highest within IS cohort.

**Fig. 2:**
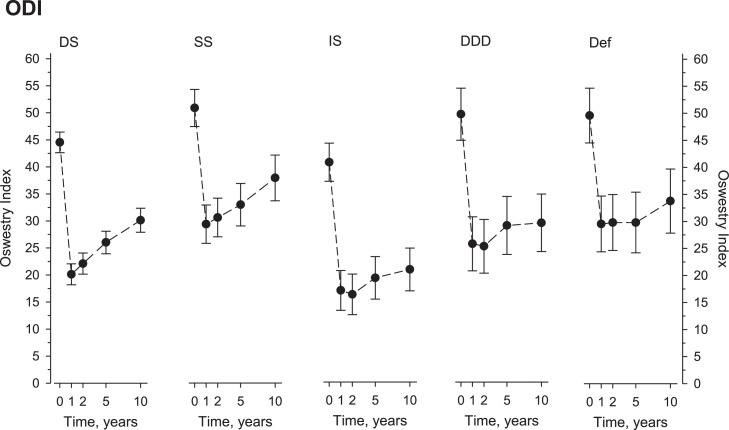
The Oswestry disability index (ODI) at baseline and follow-up according to surgical indication.

All diagnostic cohorts demonstrated significant decrease in ODI after surgery (Fig. 2), followed by at least marginal upturn over time. The new rise seemed more manifest in patients with DS or SS. In them, the 10-year change in ODI paralleled a reported minimum clinically important change (MCID) of −12.8 points [18].

**Fig. 3:**
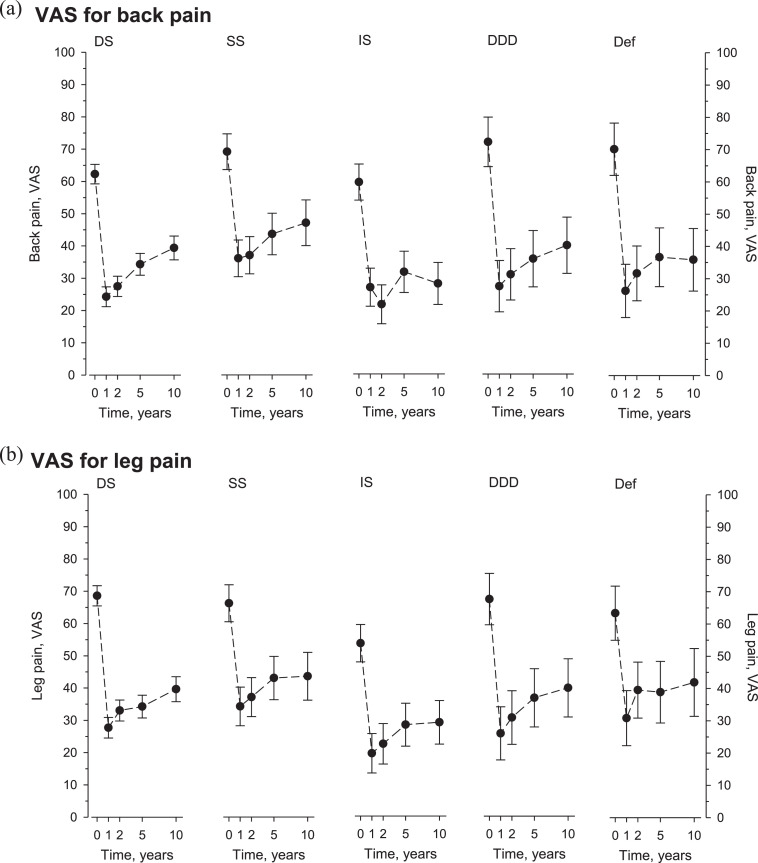
(A) Visual analogue scale (VAS) for back pain at baseline and follow-up according to surgical indication. (B) Visual analogue scale (VAS) for leg pain at baseline and follow-up according to surgical indication.

Aligned improvements in VAS for back and leg pain (surpassing reported MCIDs of −12 and 16 as extrapolated from the numeric rating scale [18]) were observed throughout follow-up (Fig. 3A and B). All cohorts except IS experienced partial recurrence of back pain, whereas radicular pain partially recurred to all groups.

**Fig. 4:**
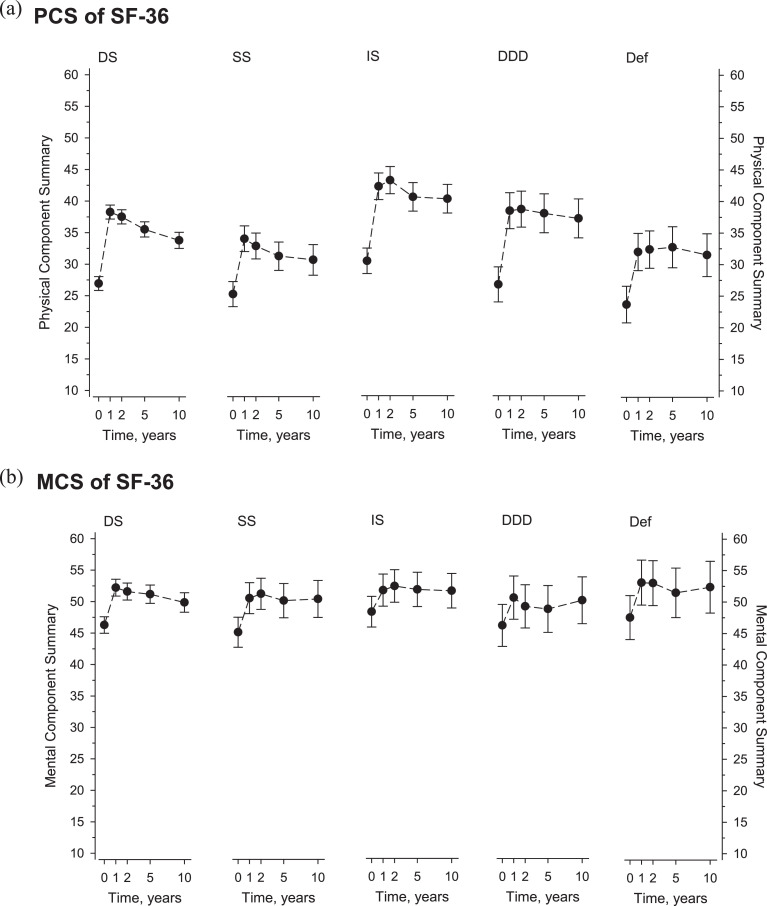
(A) The physical component summary score (PCS) of Short-Form 36 (SF-36) at baseline and follow-up according to surgical indication. B. The mental component summary score (MCS) of Short-Form 36 (SF-36) at baseline and follow-up according to surgical indication.

Significant HRQoL changes followed LSF across all diagnostic cohorts (Table 2). In the domains of SF-36, increase was greatest in physical role, and bodily pain, substantial in physical function, vitality, emotional role, social functioning, and mental health. Only general health domain prevailed negligible. Physical (PCS) and mental component summary scores (MCS) of SF-36 are presented in Fig. 4A and B. Physical component summary scores changes were greater than MCS changes. PCS changes also surpassed the reported MCID of 4.9 [18]. Minimum clinically important changes for MCS or individual domains of SF-36 have not been reported in lumbar surgery settings, authors understand. Physical component summary scores demonstrated a trend of minor loss of initial benefits over time.

**Table 2:** Mean SF-36 domains and summary scores at baseline (with SD) and their changes in follow-up (with 95% CIs) according to surgical indications.

SF-36 domains	DS N=332	SS N=102	IS N=97	DDD N=52	DF N=47
A, Physical functioning					
Baseline	30 (18)	24 (17)	45 (21)	32 (16)	23 (18)
Change at 1 y	31 (29–34)	24 (20–29)	29 (24–34)	32 (25–38)	21 (14–28)
Change at 10 y	17 (14–20)	12 (7–18)	22 (17–27)	30 (23–37)	15 (7–23)
B, Role physical					
Baseline	8 (20)	7 (18)	18 (29)	9 (21)	5 (14)
Change at 1 y	35 (31–40)	26 (17–34)	33 (25–42)	31 (20–43)	20 (8–33)
Change at 10 y	26 (21–31)	29 (19–39)	40 (31–49)	34 (21–46)	26 (12–40)
C, Bodily pain					
Baseline	27 (16)	21 (14)	29 (17)	21 (13)	22 (17)
Change at 1 y	30 (28–33)	27 (22–32)	31 (26–36)	33 (26–41)	28 (21–36)
Change at 10 years	23 (19–26)	24 (18–30)	26 (20–32)	27 (19–35)	30 (21–38)
D, General health					
Baseline	52 (18)	50 (19)	57 (22)	55 (21)	45 (19)
Change at 1 y	5 (3–7)	3 (-1 to 6)	6 (2–9)	1 (-4 to 6)	5 (0–11)
Change at 10 y	-4 (-6 to -1)	-5 (-9 to 0)	1 (-3 to 5)	-2 (-8 to 3)	0 (-6 to 6)
E, Mental health					
Baseline	64 (22)	59 (21)	66 (23)	62 (21)	63 (24)
Change at 1 y	12 (10–14)	12 (8–16)	10 (5–14)	11 (6–17)	12 (6–18)
Change at 10 y	12 (10–14)	12 (8–16)	10 (5–14)	11 (6–17)	12 (6–18)
F, Vitality					
Baseline	45 (23)	43 (20)	48 (26)	43 (23)	46 (22)
Change at 1 y	18 (16–21)	16 (12–21)	16 (11–21)	18 (11–24)	15 (9–22)
Change at 10 y	10 (8–13)	11 (6–17)	11 (6–16)	15 (8–22)	15 (8–23)
G, Social functioning					
Baseline	52 (28)	50 (29)	59 (27)	46 (26)	49 (27)
Change at 1 y	25 (22–28)	20 (15–26)	20 (15–26)	26 (18–34)	24 (16–33)
Change at 10 y	17 (14–21)	13 (6–20)	18 (12–24)	25 (16–34)	23 (14–33)
H, Role emotional					
Baseline	40 (43)	38 (42)	55 (43)	48 (44)	44 (42)
Change at 1 y	26 (21–31)	18 (8–27)	16 (6–25)	15 (2–28)	13 (-1 to 26)
Change at 10 y	14 (8–20)	20 (9–31)	21 (11–31)	14 (0–28)	11 (-5 to 26)
PCS, Physical component summary					
Baseline	27 (7)	25 (6)	30 (8)	27 (6)	24 (6)
Change at 1 y	12 (11–13)	9 (7–10)	13 (11–14)	11 (9–14)	8 (6–11)
Change at 10 y	7 (6–8)	5 (3–8)	10 (8–12)	10 (7–12)	8 (4–11)
MCS, mental component summary					
Baseline	46 (13)	44 (12)	49 (13)	45 (14)	47 (13)
Change at 1 y	6 (5–7)	6 (4–7)	3 (1–5)	5 (2–8)	5 (2–8)
Change at 10 y	3 (2–5)	6 (3–8)	3 (0–5)	4 (0–7)	4 (0–8)

DS, degenerative spondylolisthesis; SS, spinal stenosis; IS, isthmic spondylolisthesis; DDD, degenerative disc disease; DF, deformity

SD, standard deviation; CI, confidence interval; SF-36, Short form 36 health survey.

PCS is positively impacted by domains A–D and negatively by E–H. MCS is negatively impacted by A–D and positively by E–H.

## Discussion

This prospective study demonstrated clinically meaningful benefits of LSF throughout the 10-year follow-up. Early benefits observed at 1-year were partially lost later. These trends were visible in all diagnostic cohorts.

The present 10-year trajectories in ODI were consistent with prior reports of LSF outcomes for populations with various indications (mean long-term ODI change from −10 to −26) [8,^9^,^19^,20]. In their study comparing fusion techniques, Hoy et al. [19] reported 2-year benefits to be preserved at 5 to 10 years Glassman et al. [9] reported stable ODI through the 5-year follow-up of single-level fusions for various indications. Contrasting those, Maruenda et al. [8] found that after initial benefits of fusion for degenerative indications, ODI was reverted to baseline level by 10 years. High occurrence of adjacent segment disease explained this loss of benefit (25% had undergone revision surgery by 10 years, when those having undergone revision were functionally superior to the rest of patients). Also, we have previously reported a 10-year revision rate of 18% for adjacent segment disease in Tampere University Hospital [21]. The population of that study partially overlapped the present one. Accordingly, progressing spinal disease likely plays a key role in recurring symptoms.

Long-term pain score trajectories are reported more sporadically than ODI. With various indications, long-term reduction in back pain of −2.0 to −3.7 (10-point scale) and leg pain of −2.3 to −3.3 have been reported [9],20]. Our results are in line with those. Fairly aligned patterns across ODI and pain scale trajectories highlight the role pain has in functional restrictions stemming from spinal disorders. We suppose the lowest trend of recurring back pain in IS cohort relates to the unique nature of that pathology (single locus vs. wide-ranging degeneration) and also to the youngest age of that cohort. Therefore, the state of overall spinal degeneration is less advanced in that cohort. The modestly rising trend in radicular pain however seemed surprisingly aligned across cohorts.

Measuring HRQoL on par with pain and disability is preferred in outcomes studies [22]. Short Form-36 is one of the most used HRQoL measures, whereof most spine studies report its physical (PCS) and occasionally also mental (MCS) component summary scores. Individual domains are reported infrequently, yet that is increasingly recommended. Obviously, treatment effect is greatest in pain and functional scores, and less prominent in more general QoL scores [23]. Also here, domains of SF-36 related to pain and physical function (physical role, bodily pain, and physical function) demonstrated the greatest part of QoL improvement. Still, the effect on Emotional role was manifest, suggesting the role of LSF in relieving depressive symptoms potentially secondary to spinal condition [24,25]. Long-term PCS changes here were consistent with prior reports on heterogeneous populations by Glassman et al. [9] (10.1) and surpassed those reported by Owens et al. [20] (2.9–5.9). Utility of MCS with spinal disorders typically characterized by physical disability is compromised due to calculation process of MCS where low physical scores boost MSC [26]. Therefore, MCS changes in our cohorts also prevailed modest and inconclusive compared to PCS changes (Fig. 3) in consistence with prior reports [26].

### Strengths and limitations

A population-based sample, validated outcome instruments, continuity of follow-up, and logical results are the strengths of this study. The 10-year response rate of 74% constitutes a limitation typical to long-term studies [14]. However, even short-term (1-year) data coverage of some national registries is yet lower [27,28]. Occasionally, outcomes of nonrespondents are anticipated to be worse [29]. However, other reports have appraised the bias from non-respondents limited [30], [31], [32]. Age-related limitations emerge in longer follow-ups: healthy patients at baseline may get dementia and other comorbidities and may even end up in nursing homes during the follow-up, making their participation no longer feasible. Of course, the functional demands of such patients are certainly lower, as well. Also, surgical practices change along with technological advancements. Advanced interbody fusion and minimally invasive techniques have emerged after the data collecting period, but their role in improving clinical outcomes remains to be endorsed, preserving the present results valid so far.

Another limitation of this study is the lack of control cohort treated without surgery. Nevertheless, as the natural course of distinguished spinal stenosis is not favorable, [33,34] we suppose the present results largely represent an actual treatment effect. The prospective follow-up here is predominantly based on PROMs. Long-term course of degenerative spinal complaints may involve repeat surgeries, which however may restore the earlier satisfactory outcomes, at best [8]. Other musculoskeletal disorders may also impact the long-term PROM trajectories. Here, lack of detailed data on complications and reoperations during follow-up restricts interpretation of our results. Exclusion criteria of the present study (fracture, tumor, infection, neuromuscular scoliosis, and postoperative conditions) precludes external validity for those indications.

In the light of above limitations, our results best serve as a real-life evidence of long-term effects of elective LSF surgeries. It is valuable to see aligned while not similar benefits across the spectrum of LSF surgeries. Moreover, benefits were visible still at 10 years despite the fact that a significant share of patients have undergone reoperations by then [8,21]. Present results emphasize the need for careful and shared decision-making for LSF surgeries.

## Conclusion

In the present study, patients undergoing elective LSF surgeries were enrolled to a prospective 10-year follow-up. Benefits of LSF on pain, disability, and HRQoL were partially lost but still meaningful at 10 years of surgery. Magnitude and longevity of benefits showed slightly varying trends across surgical indications, although benefits were demonstrated in all diagnostic groups.

## Declarations of competing interests

One or more of the authors declare financial or professional relationships on ICMJE-NASSJ disclosure forms.
